# Attachment Status Affects Heart Rate Responses to Experimental Ostracism in Inpatients with Depression

**DOI:** 10.1371/journal.pone.0150375

**Published:** 2016-03-04

**Authors:** Jannika De Rubeis, Stefan Sütterlin, Diane Lange, Markus Pawelzik, Annette van Randenborgh, Daniela Victor, Claus Vögele

**Affiliations:** 1 Eos-Klinik für Psychotherapie, Alexianer GmbH, Münster, Germany; 2 Institute for Health and Behaviour, Research Unit INSIDE, University of Luxembourg, Esch-sur-Alzette, Luxembourg; 3 Department of Psychology, Lillehammer University College, Lillehammer, Norway; 4 Department of Psychosomatic Medicine, Division of Surgery and Clinical Neuroscience, Oslo University Hospital, Rikshospitalet, Norway; 5 University of Applied Sciences Münster, Münster, Germany; 6 Research Group on Health Psychology, University of Leuven, Leuven, Belgium; UNC Chapel Hill, UNITED STATES

## Abstract

Depression is assumed to be both a risk factor for rejection and a result of it, and as such constitutes an important factor in rejection research. Attachment theory has been applied to understand psychological disorders, such as depression, and can explain individual differences in responses to rejection. Research on autonomic nervous system activity to rejection experiences has been contradictory, with opposing strings of argumentation (activating vs. numbing). We investigated autonomic nervous system-mediated peripheral physiological responses (heart rate) to experimentally manipulated ostracism (Cyberball) in 97 depressed patients with organized (n = 52) and disorganized attachment status (n = 45). Controlling for baseline mean heart rate levels, depressed patients with disorganized attachment status responded to ostracism with significantly higher increases in heart rate than depressed patients with organized attachment status (p = .029; *ηp*^*2*^ = .051). These results suggest that attachment status may be a useful indicator of autonomic responses to perceived social threat, which in turn may affect the therapeutic process and the patient-therapist relationship.

## Introduction

Social exclusion affects peoples’ psychological functioning and behavior. More precisely, it has been found to increase aggression [1–4], to decrement self-regulation [5], to threaten feelings of self-esteem, belonging, meaning, and control [6], to lead to social withdrawal, to physiological arousal, or to prosocial behavior [3]. Social exclusion is furthermore associated with the development of psychological disorders [7]. For example, the experience of social rejection has been proposed to play a prominent role in the onset and the maintenance of depression [8]. Early experiences of rejection by parents or peers, for example, are associated with internalizing problems [9] and with insecure attachment status that increase the risk for depression [10]. Rejection later in life has also been linked to depression [11]. Furthermore, interindividual differences in perceived rejection (i.e. rejection sensitivity), and hypervigilance for signs of rejection [12] are also predictors for depression.

This association between rejection and depression appears to be reciprocal [8], as depressed individuals’ behavior has been demonstrated to elicit rejection, for example by excessive reassurance seeking [13], or by engaging in reduced eye contact [14]. Thus, depression and rejection may influence each other in terms of a vicious cycle.

In view of the potential self-perpetuating properties of depression and rejection, it is important to gain a better understanding of the factors underlying this reciprocal relationship. In the current study, one possible factor—attachment status- is investigated to deepen the understanding of this relationship. Using an experimental approach (the cyberball paradigm [15–16]), the current study investigated the role of attachment status as a moderator of the association between depression severity and cardiac reactivity after the experience of social exclusion.

Attachment status is defined as a person’s chronic pattern of relational expectations, emotions, and behaviors resulting from the internalization and memory of a particular attachment history [17]. According to attachment theory [18], the quality of attachment to the primary caregiver is formed early in life and rooted in the extent with which the infant can relate to the attachment figure as a source of security [19]. Infants create internal models of their relationship with their parents that become more and more elaborated over time. Once formed, these working models shape and explain experiences in affect, memory and attention. Though especially evident during early childhood, attachment behavior is assumed to characterize human beings “from the cradle to the grave” ([20] pp. 129–130). The attachment system can be characterized in terms of emotion-regulation processes that are activated by perceived threats (i.e. social exclusion; [18; 21]. This automatic activation in the face of attachment threat prompts a person to seek proximity to important others (i.e. figures of attachment) or their mental representations in order to restore emotional balance. When attachment figures are not sensitive, reliably available and supportive, the child cannot learn to seek proximity to regulate distress. Furthermore, they are likely to have developed negative inner working models and attachment related anxiety and avoidance (called secondary attachment strategies), that is distorted emotion regulation strategies that interfere with successful coping [22]. These attachment-related differences, therefore, determine a person’s reaction to interpersonal experiences [23]. Previous research has often relied on self-report scales to assess attachment [24]. Notwithstanding their practical utility, however, self-report measures have one very important short-coming in the face of attachment research: the use of self-report measures always involves the use of conscious thought, hence constitute a reflection of self-evaluation, that is always affected by a subjective view of oneself, that in turn, is influenced by how one wished to be/ to be seen [25–27]. In contrast, classifications using an interview protocol are based on the “evaluation of unconscious defensive processes” (p.7 in [28]). The shortcoming of self-report measures is overcome in the current study with the use of a projective picture system, the Adult Attachment Projective (AAP) [29], a construct valid measure that makes use of unconscious defensive processes. The AAP is based on four attachment categories (secure, dismissing, preoccupied and unresolved), of which the three insecure attachment categories (dismissing, preoccupied, unresolved) are of importance to the current study. These insecure attachment categories can be subsumed under organized vs. disorganized attachment (dismissing, preoccupied vs. unresolved trauma) [30–31]. The need to differentiate attachment related features in clinical samples has been postulated previously [32], especially the differences between organized and disorganized attachment status. There is confirmatory data on the distinction of these categories in the face of traumatic situations (as triggered by pictures in the AAP task) using functional magnetic resonance imaging [33].

Within the group of organized attachment, preoccupied- and dismissing attachment can be distinguished. The difference in organized attachment to socially threatening events has been the focus of previous research [23–24; 34–38]. Preoccupied attachment is characterized by a “hyperactive” attachment system [17; 39], that is oversensitive to signs of potential rejection, and shows stronger neural activation to rejection in brain regions implicated in processing social rejection (i.e., dACC, anterior insula; [40]; ACC,[36]; amygdala, [38]), more intense behavioral responses to rejection [34], greater negative emotions and lower self-esteem [41]. Attachment topics are hyper-arousing [42–43], and people dwell on current or past experiences with attachment figures. Dismissing attachment, in contrast, is characterized by a deactivating attachment system [44–45]. Such persons try to minimize or avoid difficulties related to attachment experiences [42], becoming less sensitive to signs of rejection, being less comfortable getting close to others, and using avoidance strategies to regulate attachment stress [17; 39], as shown by poorer memory of attachment related events [46–47] and less neural activation in the dACC and anterior insula [40]. Investigating autonomic reactivity in (pre) marital interactions, Roisman [48] found higher heart rates in the preoccupied attachment group as compared to the dismissing group while conversing with their partners, suggesting emotional over-involvement. Responses to social exclusion are, therefore, expected to be more pronounced in people with preoccupied attachment, as opposed to people with dismissing attachment status.

Disorganized attached children show unusual behavior patterns toward the parent when needing comfort after the stressor of a brief separation. [49]. Disorganized attachment (so called “unresolved trauma”) has been identified as an important marker for developmental risk (see [50] for review) [51], is associated with higher rates of negative life events, negative teacher-child relationship experiences in middle childhood, and low early adolescent friendship quality [52]. Childhood experiences include the individual’s failure to find support and care in the face of traumatic attachment events, such as abuse or sexual trauma, loss or parental psychopathology [26; 50; 53–59], and are characterized by exhibiting social difficulties [60–61], an unstructured expression of inner distress, and the inability to regulate this distress in social relationships [62–63]. To our knowledge, no research has investigated the role of disorganized attachment for responses to social exclusion. While disorganized attachment has received some attention in the last years [62], it has not yet been investigated in the context of social exclusion. This is mainly due to the use of self-report scales in this area, which (except [64]) make use of the 3-category (secure, avoidant, anxious) self-report measures [24].

Akin to physical pain, experiences of social rejection and exclusion may signal a significant threat to individuals’ survival [65], and there is evidence from animal lesion and human neuroimaging studies suggesting that physical and social pain overlap in their underlying neural circuitry and computational processes [66–67]. Eisenberger & Liebermann suggest that this physical–social pain circuitry might share components of a broader neural alarm system, part of which may be constituted by the autonomic nervous system (ANS) [65; 68]. This and previous research on physiological reactions to socially threatening situations [35] formed the basis for investigating autonomically mediated responses to social rejection in the current study.

The experience of being threatened is typically accompanied by an increase in Sympathetic Nervous System (SNS) activity and/or Parasympathetic Nervous System (PNS) withdrawal (Social Self-preservation theory) [69], resulting in increased physiological reactivity, and therefore increases in heart rate (HR). PNS activation can also occur, as for example in the freezing or fainting response [70], or as a result of adaptive emotion regulation [71]. Results on the effects of ostracism on ANS responses, however, are far from clear. For example, there is an ongoing debate whether rejection causes activation (e.g., [65; 72]) or numbness [73]. The former view purports that rejection causes a state of alertness to social stress, which is supported by results showing worse mood, increased arousal, and lowered self-esteem in response to rejection [74–80]. The latter approach argues that emotions are dampened following rejection, as evidenced by increased pain thresholds and pain tolerance, as well as both lower positive and negative affect following rejection, and decreased HR [73, 81–85]. Bernstein and Claypool [86] argue that the intensity and type of the reaction to social rejection (i.e. activation or numbness) depends on the severity of the experience (severity hypothesis). They posit, that ostracism is perceived as a “mild” social injury (p. 191), and that less severe ostracism experiences lead to arousal, whereas the more severe lead to numbing (i.e. future-alone paradigm). This hypothesis is supported, for example by Iffland and colleagues [87], who found increases in heart rate in participants being excluded in the cyberball game, as compared to the non-excluded control condition.

In the current study we therefore examined the cardiac response pattern (i.e.: heart rate changes) after being socially excluded in depressed patients of organized and disorganized attachment status. We hypothesized that depressed individuals with a disorganized attachment status show a stronger stress response (i.e. larger increases in HR) when exposed to a mildly ostracizing situation than depressed individuals with organized attachment status. Among the organized group furthermore, we expect the preoccupied group to react with larger increases in HR than the dismissing group.

## Materials and Methods

### Participant flow

A sample of 169 inpatients with a depressive disorder as main diagnosis (major depressive disorder, recurrent depression, dysthymia; DSM-4) and treated in the EOS-Clinic, a clinic for psychotherapy in Münster, Germany, with insecure attachment status were initially eligible for the study. Exclusion criteria were comorbid addiction disorder, or excessive substance abuse (alcohol, cannabis, multiple substances, sedatives or hypnotics), psychosis, autoimmune-thyreoiditis, personality disorders due to medical conditions (e.g., brain disorders), acute illness of the lung, hormone or heart problems (e.g. high blood pressure and its drug treatment, mitral valve prolapse, cardiac arrhythmia). Within the patient population with MDD, there were very few (n = 3 of 169 participants) with secure attachment. These patients were therefore excluded. Both, physiological and attachment data were collected from 129 patients. Twelve patients had to be excluded because of computer/ ECG problems during testing. Lastly, one person was excluded because of an unusually high resting HR (123 bpm).

### Manipulation check

The item “I was ignored” on the questionnaire following the cyberball game acted as a manipulation check to ensure that ostracism was recognized as such (Likert scale ranging from 1 (don’t agree at all) to 7 (totally agree)). The mean score was 4.24 (*SD* = 1.66). Nineteen patients failed the manipulation check (that is responded to the item “I was ignored” with 1 or 2), and were therefore excluded from the following analyses. The final sample hence comprised 97 patients.

### Participant characteristics

Descriptive data of patients at intake are displayed in Table 1. 53.6% had an organized attachment status, of which 42% had a dismissive, and 58% a preoccupied-, 46% a disorganized attachment status. The majority (31%) of the patients was married, 5% were married but separated, 30% were single, 8% were living with a partner, 7% were divorced, and 1% was widowed. The clinical documentation for 16 patients was unavailable for research purposes at the time of data collection. There were no differences in family status between the organized and disorganized attached patients (*χ*^2^ (5, N = 81) = 6.14, *p* = .293). Patients’ main diagnosis was a depressive disorder (62% with recurrent Major Depressive Disorder, 32% Major Depressive Disorder, single episode, 6% dysthymia). Fifty-eight percent of the patients were female. The most frequent comorbid diagnosis was personality disorder (37%), followed by another depressive disorder (26% of patients had a second depression diagnosis), anxiety disorder or phobic disorders (18%), and eating disorders (13%). A smaller proportion of patients had comorbid OCD (3%), somatization disorders (6%) or PTSD (9%).

**Table 1:** Participant Characteristics.

	Overall			Organized			Disorganized			t	df	p	d
	N	M	SD	N	M	SD	N	M	SD				
Age	97	41.88	14.36	52	41.54	14.73	45	42.27	14.08	-.25	95	.81	0.05
No. of prev. ambulant treatment	73	1.34	1.13	37	1.54	2.23	34	1.56	1.02	-1.54	71	.13	0.37
No. of prev. inpatient treatment	71	1.55	2.08	39	1.15	1.20	34	1.56	1.94	-.04	69	.97	0.01
No. of diagnoses	97	2.53	1.29	52	2.40	1.20	45	2.67	1.33	-.99	95	.32	0.20
BDI	95	23.69	10.34	50	21.32	9.35	45	26.33	10.85	-2.42	93	.02	0.50
GSI	95	1.17	.60	50	1.07	.56	45	1.28	.64	-1.78	93	.08	0.36

Cohen’s d describes the effect size of the difference between organized and disorganized patients. d was calculated on basis of a pooled SD. BDI: Beck Depression Inventory [88]; GSI: Global Severity Index [89].

Medication was categorized according to the second level of the ATC/DDD-Classification of the World Health Organization [90]. Sixty-nine percent of the patients received pharmacological treatment at intake, 62% of which took antidepressant medication (e.g., SSRI, MAOIs, other antidepressives).

### Experimental task

#### Ostracism Manipulation

The software Cyberball [15–16] was used for the ostracism manipulation. ‘Cyberball 3’ is a computerized ball-tossing game in which the player is presented with cartoon drawings on a computer screen. The game appears as though it is online, although it is actually fully contained within the laboratory computer. Instructions are presented on the computer screen and outline that the experimenter wants to test “the effects of practicing mental visualization on task performance”. This cover story aims to encourage the participant to practice their mental visualization skills. The participant’s character is always presented at the bottom center, only showing the hands, while three fellow players are situated in front, and on the left and on the right side. When receiving the ball from one of the players, the participant can choose to whom to throw the ball by clicking on the corresponding names that are presented on the screen. In the inclusion phase, the patient receives 1/3 of all throws, and no throws in the ostracism phase [67]. In the last phase (contemplation phase), the participant contemplates on the exclusion experience by answering questions concerning the game. Patients were asked to give a subjective rating on how many throws they received, and were given questions assessing the level of need threat [91]. On average, the inclusion phase lasted 80.73 seconds (*SD* = 12.39), the ostracism phase 81.31 seconds (*SD* = 9.66), and the contemplation phase 134.83 (*SD* = 12.93) seconds.

### Assessment of attachment status

In order to assess attachment status, the Adult Attachment Projective (AAP; [29]) was conducted. The AAP is a validated measure with high inter-rater reliability using eight contour drawings to assess narrative patterns (see [29] for an example). The first picture is neutral, followed by seven attachment scenes aiming at a gradual activation of the attachment system. The theory-derived attachment events are subdivided into “monadic” and “dyadic” scenes. For each picture presented, the individual is instructed to tell a story including the following elements: how did this scene come about? What are the people feeling or thinking? How could the story end? Each story is transcribed and coded on the basis of these verbatim narratives (see [92], [28] for a thorough presentation of the method) and classified into the different attachment categories (secure, insecure ambivalent, insecure dismissive, unresolved trauma).

The AAP has been shown to have high construct validity. Buchheim, George and West [93] found high concordance between the four attachment groups as measured on the Adult Attachment Interview [94–95] and the AAP (92% k = .89. p < .000). Inter-rater reliability showed concordance ratings of 97% when comparing 4 groups (*k* = .82; *p*>.001). Test-retest reliability showed (though in a small group with only 27 people) 89% concordance (*k* = .88; *p* < .001).

In the current study, each AAP was rated by three independent trained and certified AAP assessors. All raters underwent a 10-day training and certification program in Austria and subsequently achieved concordance ratings of at least 80% in a reliability training period within one year after the training for 3 sets of 10 cases compared to an expert rater. Final categorization was reached by consensus.

### Cardiac assessment

The electrocardiogram [96] was recorded at a 500 Hz sampling rate using two disposable electrodes attached in a bipolar configuration on opposite sides of the chest of the participant. ARTiiFACT [97] was used to extract QRS complexes, determine interbeat intervals and to detect and correct measurement artifacts in the raw ECG.

### Measures

Participants’ clinical diagnoses were determined using a structured interview (SCID I, II) [98–100]. In addition, disorder-specific questionnaires were employed including the following:

Depression symptoms were assessed using the Beck’s Depression Inventory (BDI) [88], German version: [101–102]). The BDI, a self-report measure to assess intensity of depressive symptoms and attitudes, is rated on a four-point scale. The scale has good psychometric properties with reported concurrent validity scores of .71 to .89 for different self-administered questionnaires testing depression, and test- retest reliability scores of .88. Cronbach’s alpha was calculated for the current sample with a value of alpha = .89, and is, as such comparable to other German studies [101–102].

General symptoms were assessed using the Symptom-Checklist-Revised (SCL-90- R) [103–104]. The SCL-90- R is a 90-item self-report scale, divided into 9 primary symptom dimensions, and is being used to gain an overview of patients’ symptoms and their intensity. Measured on a 5-point Likert-scale, participants were asked to rate psychological and bodily symptoms that they had experienced within the last seven days. The scale has excellent psychometric properties, with internal consistency ranging from .74 to .88 for the different subscales for inpatients, and .97 for the global scales. Test-retest reliability ranges from .69 to .92 for the different subscales, and .90 for the global scales in a German population [103–104]. Cronbach’s alpha for the current sample was .97, yielding exactly the same score as the original norm-based data from the German population [104].

Participants’ level of need threat [91] was assessed after the game using a Likert scale ranging from 1 (don’t agree at all) to 7 (totally agree). Participants were asked to rate questions concerning whether or not they felt a sense of belonging (e. g. “I felt like an outsider” (R); cronbach’s alpha = .66), control (e.g., “I felt the other players decided everything” (R); cronbach’s alpha = .54) meaningful existence (e.g., „I felt meaningless”(R); cronbach’s alpha = .79) and self-esteem (e.g., “I felt insecure” (R); cronbach’s alpha = .71). Because of low cronbach’s alpha on the control and belonging scales, these scales were omitted from the analyses.

### Procedure

The experimental session (Cyberball) was conducted in a testing room during the first week of admission to the hospital using an IBM compatible PC and displayed on a 17’ screen. The AAP was conducted within one week thereafter, but never on the same day to minimize the risk of carry-over effects of the experimental session to the AAP. Written informed consent was obtained from all participants prior to the experimental session. Ethics approval was obtained from the Ethics committee of the “Medical Association Westfalen-Lippe” prior to any data collection, including all pseudonymized clinical routine data.

Prior to the experimental session, participants were asked to refrain from smoking, drinking coffee or exercising heavily. Participants attended the experimental session individually. On arrival, the participant was seated, and basic functions of the ANS and the rational for testing it were explained. Thereafter, participants were asked to rate their health and average physical activity level (overall approx. 10–15 minutes). Transducers were then attached, followed by a five-minute monitored baseline period. Thereafter, the instructions for the cyberball game were presented on the screen. The experimenter was seated in a corner of the room to support the participant if needed, but out of the participants’ field of vision. Upon completion of the Cyberball game, participants were given further questionnaires. HR was monitored throughout the game (inclusion and ostracism phase) and while participants filled out the questionnaires (contemplation phase). At the end of the session the transducers were removed, and participants thanked for their participation, and debriefed.

### Data Analysis

An univariate ANCOVA was carried out to compare HR changes between the ostracism and contemplation phase of the cyberball game between organized and disorganized participants while controlling for baseline HR. The independent variable was the attachment status, the dependent variable was change in HR between the ostracism and the contemplation phase of the cyberball game. HR was expressed in beats-per-minute (bpm) and digitally stored on a PC for later analysis. Finally, prior to the tests of significance normality of distributions was confirmed. Lastly, HR changes between preoccupied and dismissing depressed patients were compared using the same statistical analysis as described above.

## Results

### Cardiac reaction to ostracism

**Fig 1:**
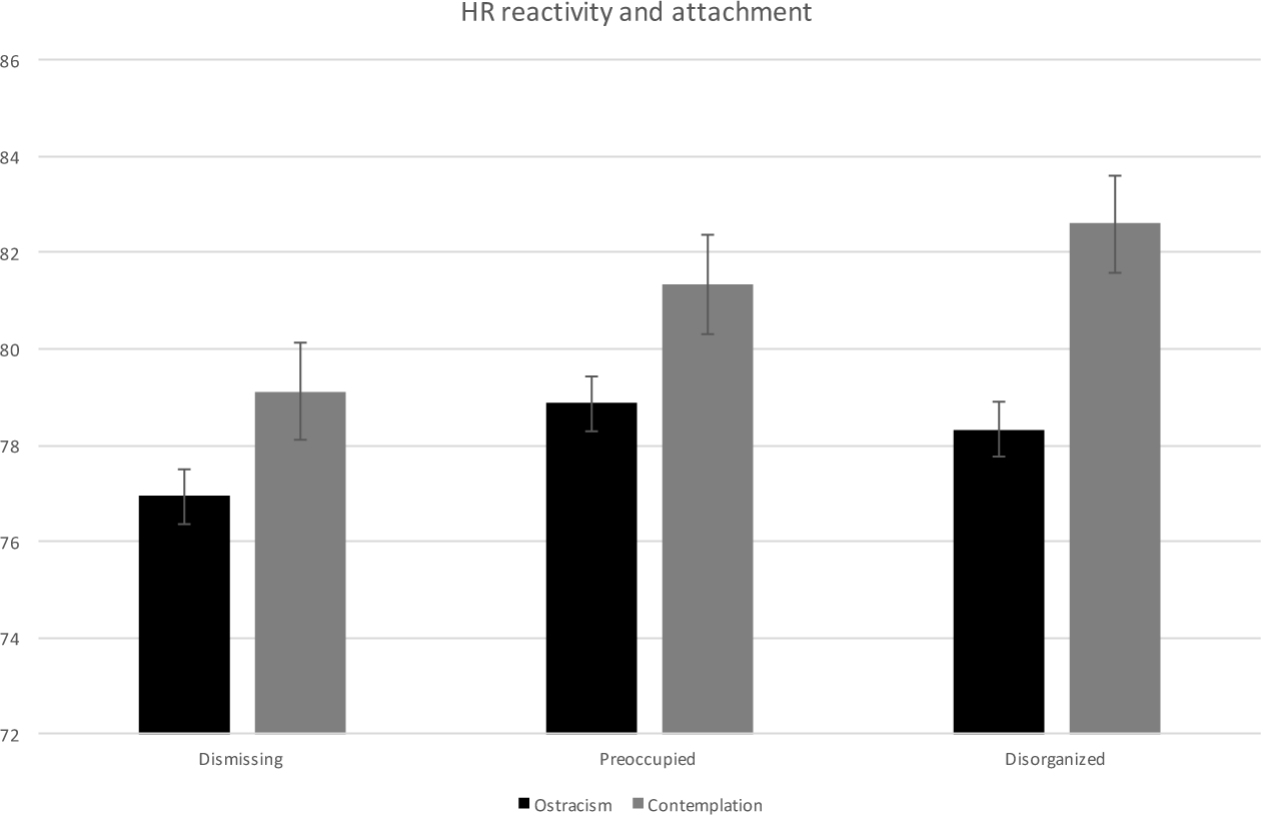
HR reactivity in dismissing, preoccupied and disorganized patients with depression.

**Fig 2:**
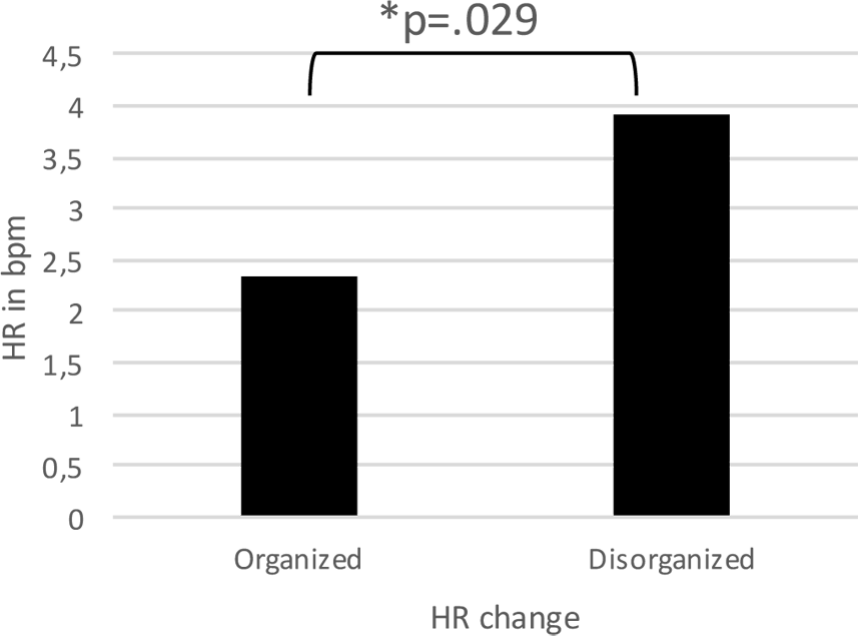
HR change in organized and disorganized patients with depression. Depressed patients with a disorganized attachment status showed significantly higher increases in HR after being ostracized even after controlling for baseline mean HR.

There was no difference in HR between the two groups (organized vs disorganized) at baseline, i.e. the 5 minute resting condition before the cyberball game (*t*(92) = -1.71, *p* = .09). A mild correlation between HR at baseline and depression severity (BDI scores) could be found in the current sample (*rtwo-tailed* = .212; *p* = .04). HR increased on average by 3.85 bpm (SD = 3.69) in the disorganized group, compared to 2.03 bpm (SD = 4.35) in the organized group (*d* = 0.43). Fig 1 displays HR reactivity for all groups. The univariate ANCOVA revealed a significant difference in heart rate changes between patients of organized and disorganized attachment controlling for baseline mean HR *F*(1) = 4.92; *p* = .029; *ηp*^*2*^ = .051 (see Fig 2). Patients with disorganized attachment showed significantly higher HR changes (i.e. faster heart beat) than organized attached patients in the contemplation phase, independent of their baseline HR. As it has been argued that using change scores might be less reliable than individual scores, we covaried the HR scores in the ostracism phase and used the HR in the contemplation phase as the outcome variable. Results stayed robust with F = 4.98; p = .028; *ηp*^2^ = .050. To account for possible effects of age and sex on HR reactivity [105–109], as well as to control for potentially confounding effects of depression severity and comorbid PD, we added these variables as covariates. This, however, did not change the pattern of results (see Table 2).

**Table 2:** ANCOVA: HR reactivity in organized vs disorganized patients.

	*F*	*df*	*p*	*ηp*^*2*^
IV: attachment (organized vs. disorganized)	4.69	1	.03	.05
Covariates				
HR at baseline	2.49	1	.12	.03
BDI at intake	1.38	1	.24	.02
PD	1.82	1	.18	.02
Age	0.01	1	.94	.00
Gender	1.40	1	.24	.02

BDI: Beck Depression Inventory [101–102]; PD: Personality Disorder.

Lastly, we analyzed the subgroup of organized patients (preoccupied vs. dismissing attachment status). Both subgroups’ HR increased on average by 2 bpm between the exclusion and contemplation phase of the cyberball game (see Fig 1 for an overview of HR reactivity for all groups). The univariate ANCOVA of HR changes between these two groups revealed no significant difference controlling for baseline HR (*F*(1) = .161; *p* = .690; *ηp*^2^ = .003). These analyses of covariance were repeated for the second hypothesis, the results of which are displayed in Table 3.

**Table 3:** ANCOVA: HR reactivity in preoccupied vs dismissing patients.

	*F*	*df*	*p*	*ηp*^*2*^
IV: attachment (preoccupied vs. dismissing)	0.29	1	.59	.01
Covariates				
HR at baseline	2.80	1	.10	.06
BDI at intake	0.30	1	.59	.01
PD	0.78	1	.38	.02
Age	0.11	1	.74	.00
Gender	2.16	1	.15	.05

BDI: Beck Depression Inventory [101–102]; PD: Personality Disorder.

### Assessment of Need Threat

The self-reported level of the threat to primary needs was calculated (meaningful existence and self-esteem). Independent t-tests revealed significant differences in meaningful existence (*t*(94) = 3.89; *p* < .001, *d* = 0.80), and self-esteem (*t*(78.79) = 2.01; *p* = .047; *d* = 0.41) after the ostracism manipulation between organized (meaningful existence: M = 4.09; SD = 1.39; Self-esteem: M = 4.84; SD = 1.31) and disorganized attachment (meaningful existence: M = 2.97; SD = 1.43; Self-esteem: M = 4.19; SD = 1.81). On both scales, patients with an organized attachment status scored higher. There were no differences in either of the need threat scales between dismissing and preoccupied attached patients (smallest *p* = .31). Means and Standard deviations are displayed in Table 4.

**Table 4:** Dismissing, Preoccupied and disorganized attachment: Need Threat Means and Standard Deviations after Being Ostracized.

	Self-esteem		Meaningful Existence	
	M	SD	M	SD
Dismissing	4.68	1.44	4.32	1.42
Preoccupied	4.96	1.21	3.92	1.35
Disorganized	4.19	1.81	2.97	1.43

Lower scores index less need satisfaction (i.e., more need threat).

## Discussion

This study investigated depressed inpatients’ cardiac reactivity after experienced social exclusion in relation to their organized or disorganized attachment status. We hypothesized that participants with a disorganized attachment status would show a more pronounced cardiac acceleration after being ostracized, compared to participants with organized attachment status. The present results confirm this hypothesis, in that HR changes were significantly higher in disorganized patients, even when controlling for age, gender and comorbid personality disorders. The results support Bernstein and Claypool’s severity hypothesis [86] on the direction of emotional reactivity, which contends that ostracism increases subsequent arousal. Attachment theory posits that early attachment experience plays an especially salient role in shaping developmental adaptation [110] and development of psychopathology. The mental representations rooted in the experiences between the child and the primary caregiver influence the later regulatory pattern (behavioral, emotional, cognitive, and physiological) in the face of stressors. In other words, the attachment relationship between child and caregiver gives rise to emotion regulation strategies, that can later develop to become risk factors for psychopathology [53; 58]). Therefore, the current results support the previously postulated hypothesis [111] that attachment theory could be extended to a biopsychosocial model of health that in turn, can explain some aspects of vulnerability to ill-health and gives rise to a range of hypotheses concerning behavior, cognition and affect. More precisely, (disorganized) attachment is hypothesized to form a diathesis that contributes to the (physiological) responses to social stress in individuals. The results are in line with our assumption that disorganization is associated with greater physiological arousal after perceived exclusion. Disorganized attached individuals lack adaptive emotion regulation skills [112], which promotes higher physiological arousal in a challenging situation. For patients with disorganized attachment status, a brief period of ostracism may activate negative self-schemata, leading to prolonged arousal and problems in self-regulation [113–115]. This (over-) interpretation of a minimal rejection episode as threatening one’s self, is likely to reinforce and strengthen the negative beliefs these patients hold about relationships [53], therefore increasing their sensitivity to exclusion experiences in future social interactions. Previous research [116] supports this notion of exacerbated perception of social cues of inclusion or exclusion, in that ostracism has the capacity to enhance the categorical (e.g., “all or nothing”) perception of social information. This forms a vicious circle, that may lead to a lowered, perceived threat threshold, which in turn gives rise to and reinforces the negative schemata the individual holds about him/herself, the world and the future [117]. Knowledge about the attachment status and incorporating strategies to buffer these consequences into the therapeutic processes may be helpful [118]. More precisely, this could be done by conveying information about the attachment system, challenging deep-seated schemata (e.g., through Socratic questioning), and helping to regulate physiological arousal (e.g., biofeedback techniques).

Patients with organized attachment status responded to ostracism with higher scores in need threat dimensions (self-esteem, meaningful existence) compared to disorganized patients. Disorganized participants reacted therefore, akin to their HR change, stronger, that is with more need threat, than organized participants. This outlines, as postulated above, that this exposure to a brief period of ostracism might activate negative self-schema in disorganized participants with depression; they seem to be more fragile in their self-esteem and their feeling of meaningful existence, than organized patients with depression. These group differences in responding to social rejection further underscore the importance of the assessment of attachment status for a better understanding of the effects of ostracism in clinical groups and dictate the importance of understanding the need-fragility involved with disorganized attachment. The need threat questionnaire may form a useful tool to guide the tailoring of subsequent therapeutic strategies. such as specific approaches to foster self-esteem [119]. Even though the current study offer support for the notion that attachment affects the intensity of physiological responses, and that it can discriminate in a clinical group beyond a diagnostic category, it falls short in assessing the duration of a stress response. Future research should investigate whether, akin to a more intense stress response, it is also longer in duration. Similarly, the behavioral response was not assessed in the current study. Future studies may, next to the physiological response to social stress, investigate is behavioral counterpart, as attachment has been previously hypothesized to influence the degree to which social contact is sought. This may be especially interesting in the face of rejection experiences.

Intriguingly, the second postulated hypothesis could not be confirmed. There were no differences in heart rate changes between patients with dismissing and preoccupied attachment styles. Both responded with increases in heart rate. These findings are especially notable as dismissing attachment is typically associated with low levels of subjective distress [19]. Previous research has posed the question as to whether this is due to the failure to experience subjective distress or the failure to report it [120]. Current results support the hypothesis, that people with dismissing attachment status are just as distressed (i.e. show accelerated HR) as those with a preoccupied attachment. This notion is strengthened by reports of increased cardiac reactivity in avoidant infants in the strange situation paradigm: Sroufe and Waters [121] showed in an early study accelerated heart rate in both dismissing and preoccupied children, while only dismissing attached children minimized the display of emotion. More recently, Hill-Soderlund and colleagues [122], comparing dismissing children with secure ones, found higher vagal withdrawal in dismissing children, indicating greater allostatic load. Similarly, [48; 113; 123–124] heightened cardiac reactivity was found in participants using deactivating (as seen in dismissing attachment) and hyper-activating (as seen in preoccupied attachment) strategies when asked to recall rejection experiences from their parents. Furthermore, investigating compensatory effects related to attachment, Jain & Labouvie-Vief [125] found attenuation effects of attachment anxiety on HR activation on the one hand, and higher activation than expected in dismissing individuals on the other hand. The researchers argue that anxious individuals have relatively congruent perceptions of their inner state [113; 126], and that lower levels of arousal may facilitate self-regulation, especially compared to those with high attachment avoidance. While their research shows dismissing individuals to have generally lower arousal levels, they argue that the effectiveness of inhibiting attachment related information vanishes under more difficult task conditions [127–128]. That is, under conditions of cognitive overload, the arousal level of dismissing individuals rises to that of anxious ones. In a similar vein, Ehrenthal and colleagues [129] argue that the delayed blood pressure recovery in dismissing individuals is related to more negative emotion, for example, anger and to dysfunctional cognitions. Contrary to our hypothesis, the current results suggest that both individuals with dismissing or preoccupied attachment styles respond similarly with HR increases in the face of rejection. Even though dismissing individuals aim at minimizing or avoiding difficulties related to attachment experiences [42], this is not accompanied by reduced cardiac arousal. These findings provide further support for the social pain theory, and the ubiquitousness of social pain [65–68]. Future research should investigate this lack of correspondence between overt behavior and autonomic responses to social exclusion in dismissing attached persons [130]. While it has been previously shown that attachment insecurity is associated with poorer emotion regulation strategies [120], more research is needed to better understand emotion regulation processes, behavioral strategies and its physiological correlates.

Furthermore, it is important to note, that the construct “attachment style” as measured by self-report scales and “attachment status” as defined by the Bowlby-Ainsworth construct have been found to differ from each other [131] as there does not seem to be any correspondence between self-report measures and the “gold-standard” of attachment research [28], the Adult Attachment Interview (AAI, [132; 25; 131]. Attachment style, as measured by self-report questionnaires is a product of thoughts that enter an individual’s consciousness whereas developmental attachment patterns assessed by narrative techniques (such as the AAP) are based on the evaluation of defensive processes, and therefore are in their method closer to the original, strange situation paradigm. Furthermore, its validity and reliability have been well established [29; 133]. The results need to be interpreted with caution when comparing them to other studies that have used self-report scales. The distribution we found in our sample was similar to that of the validation study using the AAP in a large community sample [134]. In a sample of 119 insecure participants, the researchers found 37% to be dismissing, 25% preoccupied and 44% unresolved. Within our solely clinical sample, we found a comparable number of unresolved attachment (46%); however, a smaller number of dismissing (23%), and a larger number of preoccupied attachment (31%). We hypothesize that individuals with a dismissing attachment status do not seek treatment as readily as those with other attachment strategies [111], rather than this difference being due to a lower number of depressed individuals in this attachment category. Future research should investigate this hypothesis of treatment seeking and dismissing attachment. We did not include any securely attached participants, as these represented only a small minority in the current inpatient sample (3 of 169 = 2%, whereas George and West report 17% in their sample of n = 144). This gives further support for the buffering effect of attachment security on psychopathology [135].

The present results show that attachment is important for understanding cardiac-autonomic reactivity to social stress. From a clinical perspective the present results suggest that attachment status may be a useful indicator of responses to (perceived) social threat, which in turn may affect the therapeutic process as well as the patient-therapist relationship. Furthermore, coping with social stress ought to constitute a significant proportion of the therapeutic work for disorganized patients in particular, concomitant to the augmentation of positive activities in order to heighten the reinforcement balance. Training of social competencies is a *conditio-sine-qua-non* in the treatment of depression, either to resolve social deficits, or to improve social competencies so that social exclusion can be avoided or reduced. During therapeutic work, rejection sensitivity, just as coping strategies to actual or perceived social rejection should form part of the therapeutic process. One therapeutic goal may be patient’s improved understanding of how their rejection sensitivity affects daily activities and responses to social exclusion.

It has been previously shown that perseverative cognition, as manifested in rumination or worry, is a common stress response [136] that can extend the physiological stress response [81]. The here presented results can thus be interpreted in terms of the rumination arousal model [136]. As previously postulated, people with a disorganized attachment status may exhibit distorted emotion regulation strategies, that interfere with successful coping [22]. One of these strategies could be rumination. The current results therefore suggest that future research should investigate cognitive processes after ostracism for a better understanding of the psychological correlates of the observed persisting physiological arousal. Although one limitation of this study was the presence of a variety of comorbid mental conditions, this may also represent an opportunity: a transdiagnostic approach may portray the importance of underlying factors, such as attachment status. Although we only tested patients with a clinical diagnosis of depression, future work may be able to include larger groups of patients across a variety of mental disorders and examine whether patients could be classifiable based on their attachment status.

Buchheim and colleagues [32] underline the importance of comparing organized and disorganized attachment status in depressed patients, acknowledging the complexity of depression and attachment and the lack of previous research. Extending the findings by analyzing differences between each attachment status separately, or between secure and insecure attachment representations will further portraying the picture of autonomic reactions to social stress in the different attachment representations. Because trauma was not assessed here, we cannot rule out that severity of trauma may account for the results of disorganization on heartrate and recommend that it be studied in future work.

A considerable amount of literature suggests furthermore that lower physiological reactivity is related to a more expressive and externalizing regulation style, whereas heightened reactivity is related to an internalizing or inhibiting emotion regulation style (e.g., [137–140]. The behavioral strategy in the face of social exclusion should be followed up by making use of narrative techniques to measure attachment (AAI or AAP). More precisely, it remains to be investigated whether disorganized attachment is indeed (as suggested by the current findings in light of the research outline above [137–140]) associated with internalizing emotion regulation strategies. Meta-analytic evidence [59] rather suggests, that the association between insecure attachment and internalizing strategies is rather small, and the effect solely explained by avoidant (i.e. dismissing) attachment. Disorganization, as shown in a similar meta-analysis [58], is rather associated with externalizing symptoms. These unclear, even opposing results should be investigated in adult populations (where research has been scarce), making use of the AAP or AAI.

### Limitations

A first limitation of this study concerns the lack of pre-post cyberball game mood ratings. Although not an imminent part of the research question, self-reported data on emotional experience and the investigation of discrepancies between these and physiological markers of arousal could have provided additional insights. Secondly, due to the lack of documentation at the time of data collection, we could not report any inter-rater reliability of the AAP ratings of the three independent raters.

Furthermore, not enough data was available to include the comparison of secure vs. insecure attachment representations, because of the low number of securely attached patients in the current sample. Future research should investigate attachment and responses to social exclusion with healthy participants, participants with different diagnoses, in order to better understand the link between attachment and responses to social rejection.

## Conclusions

Social exclusion evoked a physiological response pattern that was–as hypothesized- characterized by heart rate increases. Depressed patients with a disorganized attachment status reacted with a more pronounced hear rate acceleration than their organized counterparts, supporting the difficulty of this patient group in regulating emotions and supports the hypothesis of disorganized attachment being a risk factor for ongoing or a relapse to depression after being socially excluded.

## Supporting Information

S1 Dataset Attachment status and ostracism in inpatients with depression. (SAV)
